# Development and Validation of AI System for Tooth Detection and Diagnosis in Dental Radiographs

**DOI:** 10.1016/j.identj.2026.109576

**Published:** 2026-04-26

**Authors:** Niels van Nistelrooij, Peter Jurkáček, Julian Runge, Wesley Do, Khalid El Ghoul, Tong Xi, Maximiliano Sergio Cenci, Bas A.C. Loomans, Shankeeth Vinayahalingam

**Affiliations:** a Department of Oral and Maxillofacial Surgery, Radboud University Medical Center, Nijmegen, The Netherlands; b AID s.r.o., Bratislava, Slovakia; c Department of Oral, Maxillofacial, and Plastic Facial Surgery, University Hospital Münster, Münster, Germany; d Department of Oral and Maxillofacial Surgery, Erasmus University Medical Center, Rotterdam, The Netherlands; e Department of Dentistry, Radboud University Medical Center, Nijmegen, The Netherlands

**Keywords:** Artificial intelligence, Bitewing radiography, Dental caries, Mixed dentition, Panoramic radiography, Primary teeth

## Abstract

**Introduction and Aims:**

To develop and validate an AI-automated system for dental charting that accounts for multiple radiograph types and younger patients.

**Methods:**

A total of 3705 dental radiographs were collected in Slovakia and Egypt between 2023 and 2024, including orthopantomograms (OPGs) and intraoral radiographs (e.g. bitewings). Complete teeth were manually annotated with bounding boxes and tooth numbers by 2 calibrated annotators. Subsequently, teeth were assessed by at least 3 trained clinicians (10 total, 2-13 years of clinical experience) for the presence of ten dental findings (caries, crown, filling, implant, pontic, periapical lesion, primary tooth, retained root, root canal treatment, unerupted tooth). Assessments were aggregated using the Dawid-Skene model. The AI system comprised 3 deep learning stages for modality classification (OPG or intraoral radiograph; EfficientNetV2), tooth detection per modality (RTMDet), and dental finding classification (EfficientNetV2) and was evaluated with 5-fold cross-validation on 400 held-out radiographs against the clinicians and 3 independent dentists.

**Results:**

Tooth detection was highly effective (F1-score: OPG = 0.99, intraoral = 0.98; tooth number accuracy: OPG = 0.98, intraoral = 0.96) with decreased effectiveness for primary teeth. Dental finding classification saw mixed effectiveness (F1-score: 0.52 to 0.99) with a lower effectiveness for disease-related findings. The system was more accurate than the dentists for caries, crowns, fillings, primary teeth, root canal treatments, and unerupted teeth.

**Conclusion:**

The AI system outperformed dentists for common findings, but more radiographs and annotations are required to effectively interpret rare conditions. Overall, it can support radiographic diagnosis and speed up dental charting for most patients.

**Clinical Relevance:**

Interpretation of dental radiographs requires clinical experience and remains challenging. An effective AI system was developed for tooth detection and diagnosis in several types of dental radiographs of young and adult patients. Its findings can enhance the clinical workflow, facilitate dentist-patient communication, and improve diagnostic consistency for prevalent findings.

## Introduction

Dental radiographs are used to image dental hard tissues during regular checkups and are routinely used for documentation, diagnosis, monitoring, and treatment planning.^1^^,^^2^ A dental radiograph can be acquired extraorally or intraorally, depending on the imaging technique.^3^ The extraoral orthopantomogram (OPG) provides a panoramic overview of the oral and maxillofacial region,^4^ whereas intraoral radiographs capture a smaller area (3 × 4 cm) with a higher resolution in several common locations. For example, a bitewing shows the crowns of posterior teeth, whereas a periapical (PA) radiograph displays both the crowns and roots of maxillary or mandibular teeth.^5^

Effective interpretation of dental radiographs is challenging and requires clinical experience, while it remains operator-dependent, somewhat unreliable, and time-consuming, even following training.^6^, ^7^, ^8^ For example, caries lesions are mostly diagnosed using bitewings where the interpretation may miss initial enamel lesions or confuse a radiolucent dental restoration for a caries lesion.^9^ Furthermore, documenting a patient’s dental status to complete their patient records, such as missing teeth, fillings, crowns, implants, and bridges, is a diligent process that is repeated for every radiograph, particularly for younger patients with a mixed dentition.^10^ Interpreting radiographs of mixed dentitions is especially challenging, due to superimposition of primary and permanent teeth and errors in patient positioning.^11^

Artificial intelligence (AI), particularly deep learning, is extensively researched for the support and automation of common clinical tasks, including radiograph interpretation.^12^, ^13^, ^14^ AI systems learn patterns and features to replicate the reference data^15^ and can support the detection, segmentation, and classification of teeth in dental radiographs.^16^ Furthermore, AI systems have been proposed for the diagnosis of caries lesions in bitewings^17^ and periapical lesions in PAs,^18^ showing promising results compared to experts. Recent approaches utilizing OPGs proposed automated methods for the identification of peri-implantitis,^19^ the analysis of potentially ectopic eruptions,^20^ and the automated diagnosis of periodontitis.^21^

However, only a few AI systems have been proposed for interpreting all types of 2-dimensional dental radiographs,^22^ as most studies focus on a single radiograph type. In contrast, commercial AI systems for interpreting dental radiographs do encompass multiple radiograph types, though their diagnostic accuracy for patients with mixed dentitions is rarely reported. Furthermore, most commercial systems provide predictions without explanation. Without explanations, dentists are more susceptible to automation bias, as they cannot question the system’s findings, leading to unwarranted trust and overreliance on the system.^23^ AI systems reported in the literature are rarely validated against dental practitioners, instead only evaluated against ground-truth annotations.^24^ This limits the interpretation of whether the results achieved by recent AI systems are clinically acceptable, as a direct comparison of the AI system to dentists is not available.

Therefore, the current study aimed to develop and validate an AI system for dental charting of different types of dental radiographs for mixed and permanent dentitions. The null hypothesis was that the diagnostic accuracy achieved by the AI system was less than or equal to that of an average dentist in the detection of prevalent findings (> 5% of images) in dental radiographs.

## Material and methods

This study followed the ethical principles of the Word Medical Association (Declaration of Helsinki), and ethical approval was acquired for the use of patient data from the institutional review board (METC Oost-Nederland, file number 2024-17715). Each patient submitted their informed consent, and all data were pseudonymized before analysis. The checklist for AI research in dentistry was consulted.^25^

### Data

3705 dental radiographs from 2094 patients were retrospectively collected from Andel Elite Dental Center, Hlohovec, Slovakia and La Clinique, Alexandria, Egypt, between 2023 and 2024 using convenience sampling. The radiographs were acquired during regular dental checkups and standard radiographic protocols, approved locally before the start of the current study, were followed while using the imaging equipment available at the dental clinics. Images from Slovakia were collected using CLINIVIEW™ (DEXIS) and NNT (Newtom), whereas images from Egypt were collected using Romexis (Planmeca). All collected images were validated, securely stored, and finally exported in JPEG or PNG format.

The patient sample represented a wide age range, including children, as well as several ethnicities (Table 1). The most common dental radiograph types were the OPG (n = 1557) and bitewing (n = 1512), followed by the periapical radiograph (n = 451) and other radiographs (n = 185), including occlusal and pediatric radiographs. The median size of OPGs was 2432 by 1280 pixels and the median size of intraoral radiographs was 1324 by 1029 pixels. Criteria for excluding images followed clinical guidelines, such as images that were inverted, had severe imaging artifacts, or those with an ambiguous radiograph type.

**Table 1:** Characteristics of the 2094 included patients in terms of gender, age, and ethnicity and the characteristics of the dental findings included in this study. The number of teeth and prevalence per image is reported for teeth with an aggregated reader score above 0.5. Each distribution is determined for aggregated reader scores above 0.1 with the score on the x-axis and the number of teeth on the y-axis. 59,810 teeth were assessed by at least 3 clinicians for the presence of ten dental findings.

### Data annotation

The annotation process was performed in 2 rounds, each preceded by a training or calibration phase. Annotators were only provided the dental radiographs per patient, without any patient information.

For the first calibration phase, a subset of images was taken and the complete teeth were manually annotated with bounding boxes and tooth numbers following the FDI tooth numbering system.^26^ These initial annotations were reviewed by and jointly discussed with a dental professional to derive a consensus on the annotation procedure. Subsequently, teeth were annotated in all images by a professional annotator using Computer Vision Annotation Tool (CVAT.ai Corporation) and reviewed and revised by a second data annotator.

The second round of the annotation process was based on the tooth annotations of the first round and assessed the presence of ten radiographic findings per tooth (Supplementary Table 1). Prior to performing the annotations, all potential clinicians underwent a training session to ensure consistent interpretation of dental findings. During this session, a number of images were independently assessed and jointly discussed until consensus was reached. Of all participants, ten clinicians that achieved a good agreement with the consensus and with at least 2 years of clinical experience were included for this study. The first-round annotations were not performed by any included clinician.

For the second round of annotations, each annotated tooth was assessed by at least 3 clinicians independently and the clinician assessments (present/absent) were aggregated using the Dawid-Skene model.^27^ This is a probabilistic method that estimates the presence of a dental finding by weighting each clinician’s assessment according to their inferred diagnostic accuracy. The model determined an *aggregated reader score* between 0 and 1, where 0 indicated absolute certainty that the dental finding was absent and 1 indicated absolute certainty that it was present. Compared to other aggregation methods such as majority voting or average voting, the Dawid-Skene model results in more confident scores, automatically weighted by each annotator’s reliability.

To validate the AI system against dentists, 3 dentists not previously employed as clinicians were included with varying levels of clinical experience (JR: dentist, 5 years of clinical experience; WD and KEG: residents in oral and maxillofacial surgery, 8 years of clinical experience). The dentists were provided with bounding boxes around all included teeth on the annotation platform Darwin (V7). They were instructed to tag six dental findings (implant, crown, pontic, unerupted tooth, retained root, primary tooth), segment 4 dental findings (filling, root canal treatment, periapical lesion, caries lesion), and match the segmentations to the corresponding tooth bounding boxes.

### Deep learning model

**Fig. 1:**
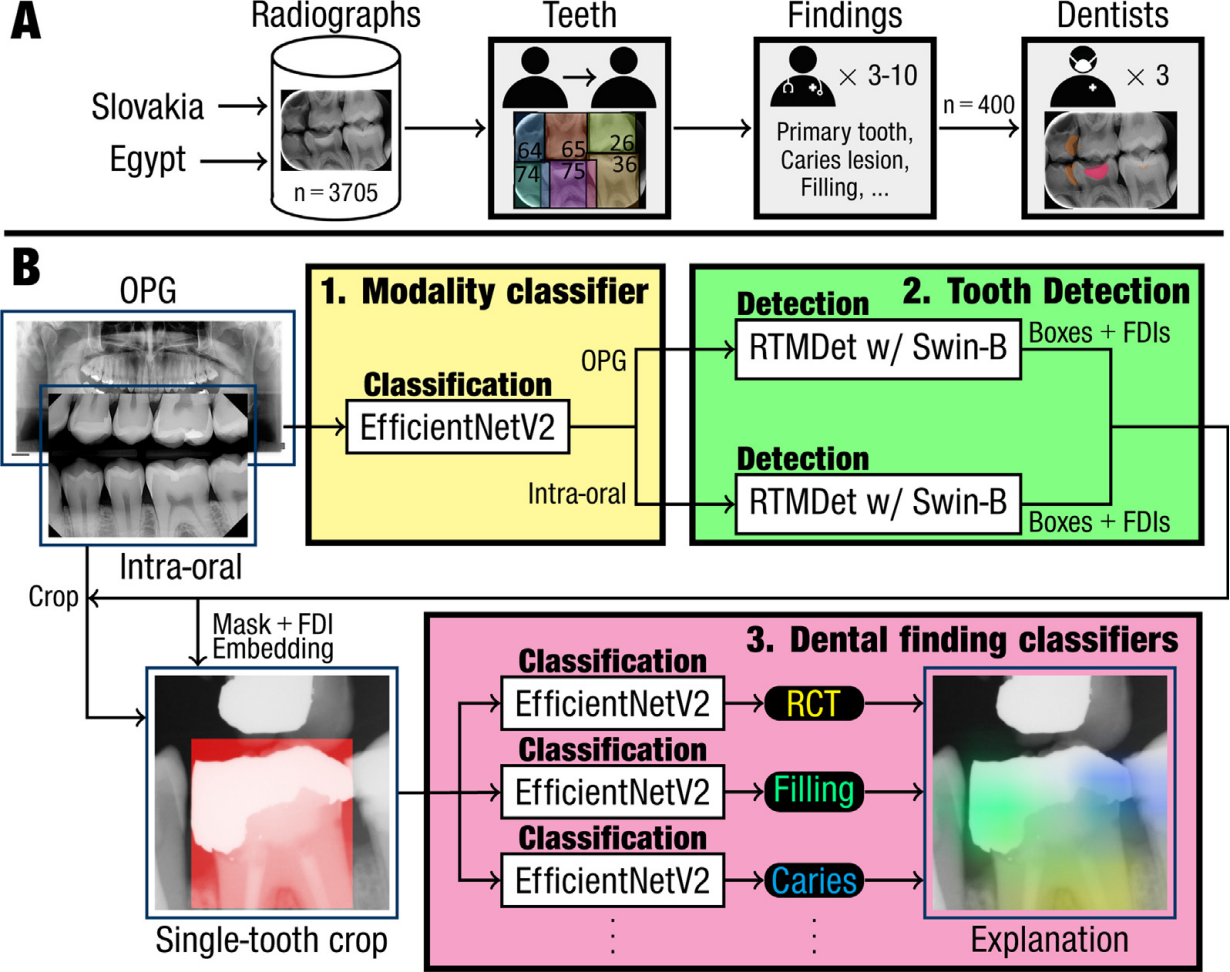
(A) Flow chart of data collection and data annotations. 3,705 dental radiographs were collected from 2 clinical centers in Slovakia and Egypt and the complete teeth were annotated with bounding boxes and FDI labels by 2 annotators in succession. Based on the annotated teeth, ten dental findings were assessed per tooth by 3 to 10 clinicians. Finally, 400 radiographs were selected for validation and examined by 3 independent dentists to establish an average diagnostic accuracy. (B) Overview of deep learning system comprising 3 stages. The first stage predicts the image modality (extraoral, intraoral), followed by a modality-specific tooth detection and FDI numbering model. Each detected tooth is cropped from the original radiograph and processed by dental finding classifiers to predict the presence of ten dental findings. The predicted findings could be explained by Grad-CAM to show which pixels contributed most to the positive predictions.^31^ RCT = root canal treatment.

A fully automated system for interpreting dental radiographs was developed comprising 3 stages: modality classification, tooth detection, and dental finding classification. See Figure 1 for an overview and Supplementary Section 1 for details on model training.

*Modality classification.* A radiograph was categorized as extraoral (OPG) or intraoral (bitewing, PA, other) using a binary classification model based on the EfficientNetV2 architecture.^28^

*Tooth detection.* The images of each radiograph modality (extraoral, intraoral) were used to develop 2 modality-specific tooth detection models. The task of tooth detection was 2-fold: localize each tooth with a bounding box and label the tooth with an FDI number. As the patients included both primary and mixed dentitions, the tooth detection models predicted 52 FDI numbers (eight permanent and 5 primary teeth per quadrant). These models were implemented using the RTMDet architecture^29^ with a Swin-B backbone network.^30^

*Dental finding classification.* The third stage processed single-tooth crops from a radiograph to assess the presence of dental findings. As a tooth could have multiple findings (e.g. implant + crown), this stage needed to consider multiple labels simultaneously. Therefore, the assessment of each finding was implemented using a separate binary classification model, where predictions from all models were combined to determine a multi-label. Each model was trained using all radiograph types, as the differences in projection and resolution had little effect on the single-tooth crops.

To prepare a single-tooth crop, a square region of interest around the tooth’s bounding box was determined. A second image channel was added, highlighting the original bounding box in the crop to signify the tooth that should be assessed. Furthermore, the tooth’s FDI number was converted to an input vector by encoding the dental arch (upper, lower) and the tooth’s position within its quadrant. This input vector was processed by a multi-layer perceptron (MLP) to determine an FDI embedding. Finally, the FDI embedding was concatenated with the image features from the backbone network, and a fully connected layer predicted the presence of a dental finding. The same model architecture as for the modality classifier was used, and each model was supervised using the cross-entropy loss with the aggregated reader score as target to promote generalizability by calibrating the model’s confidence more effectively.^31^

*Model inference.* By applying a dental radiograph to the 3 stages in succession, the deep learning system could predict the image’s modality, teeth, FDI numbers, and zero or more dental findings per tooth. Furthermore, as the assessment of each dental finding was implemented in a separate model, a class activation map (CAM) was used to explain the predicted dental findings. More specifically, Grad-CAM was used to determine how much each pixel contributed to a positive prediction, when the model predicted a finding to be present.^32^ This technique highlighted the areas of the single-tooth crop that the model paid attention to, enabling a better understanding of which features the model used for its assessment.

### Evaluation and comparison

The developed system was evaluated using held-out testing, where 5-fold cross-validation was applied to the remaining data. More specifically, a selection of 242 held-out patients with 200 OPGs, 150 bitewings, and 50 PAs was determined with stratification based on image modality, age group, gender, ethnicity, FDI numbers, and dental findings using Scikit-Multilearn (v. 0.2.0).^33^ To ensure a reliable evaluation, held-out radiographs were assessed by at least 4 clinicians. The remaining patients were divided over 5 folds. Each model was trained 5 times, using patient data from 4 folds, and evaluated using the held-out patients. By utilizing both held-out testing and cross-validation, both the effectiveness and reliability of each stage for the held-out patients could be determined.

The dental finding classifiers were compared to the dentists, after each dentist independently annotated the dental findings in all 400 held-out radiographs. To compare the model predictions and dentist annotations to the clinicians, each aggregated reader score above 0.5 was considered indicative of the presence of a finding. Furthermore, the dentist segmentations were superimposed and compared to the Grad-CAM explanations.

### Statistical analysis

Statistical analyses were performed using Scikit-learn (v. 1.5.2).^34^ Modality classification was evaluated with OPG as the positive class using accuracy $=\frac{TP+TN}{TP+FP+FN+TN}$ with TP true-positives, TN true-negatives, FP false-positives, and FN false-negatives. The effectiveness of the tooth detection models was investigated using F1-score $=\frac{2TP}{2TP+FP+FN}$, where annotated and predicted teeth were matched with an intersection over union (IoU) of at least 0.5. FDI numbering was evaluated by comparing the labels of matched teeth using accuracy, and the overall effectiveness was measured using mean average precision (mAP). The dental finding classifiers and dentists were evaluated against the clinicians using support =TP+FN, precision $=\frac{TP}{TP+FP}$, sensitivity $=\frac{TP}{TP+FN}$, specificity $=\frac{TN}{TN+FP}$, accuracy, and F1-score. Furthermore, receiver-operating-characteristics (ROC) curves were determined based on the results of the AI system compared to the clinicians. These ROC curves were used to compute the area under the ROC curve (AUC) and to compare the AI system’s effectiveness to the operating points of the dentists.

## Results

The dental findings included in this study are shown in Table 1. The distributions of their aggregated reader scores show some scores between 0.1 and 0.9 for dental findings related to disease (caries lesion, periapical lesion, retained root), meaning that the presence or absence of a dental finding was not absolutely certain.

Results for modality classification revealed an accuracy of 1.0.

Evaluation of tooth detection and FDI numbering saw a higher effectiveness for OPGs (Tooth F1 = 0.989, FDI accuracy = 0.980) than for intraoral radiographs (Tooth F1 = 0.978, FDI accuracy = 0.956) (Table 2). However, the overall effectiveness of the AI system was higher for intraoral radiographs (mAP = 0.869) than for OPGs (mAP = 0.779). Furthermore, the results for primary teeth (n = 307) were less effective compared to the results for permanent teeth (n = 7112).

**Table 2:** Tooth detection and numbering results for the held-out radiographs.

Modality (n)	Teeth	Support	Tooth F1	FDI Accuracy	mAP@50	mAP@75	mAP@50-95
Extraoral (n=200)	Permanent	5631	0.991	0.980	0.990	0.969	0.875
	Primary	241	0.945	0.978	0.844	0.690	0.625
	All	5872	0.989	0.980	0.934	0.861	0.779
Intraoral (n=200)	Permanent	1481	0.979	0.957	0.940	0.920	0.859
	Primary	66	0.962	0.947	0.971	0.962	0.895
	All	1547	0.978	0.956	0.949	0.932	0.869

All metrics are reported as mean over 5 cross-validation models. mAP@x denotes mean average precision where detected and reference teeth are matched with intersection over union (IoU) threshold(s) x.

The results of dental finding classification in Table 3 revealed a higher effectiveness for tooth restorations, tooth replacements, and tooth eruption stages with F1-scores above 0.9 compared to dental findings related to disease with F1-scores ranging from 0.52 to 0.83. More specifically, the AI system achieved F1-scores for crowns, fillings, implants, pontics, root canal treatments, primary teeth, unerupted teeth, caries lesions, PA lesions, and retained roots of 0.93, 0.94, 0.97, 0.95, 0.98, 0.99, 0.96, 0.71, 0.52, and 0.83, respectively, as well as AUCs of 0.997, 0.994, 1.000, 0.997, 1.000, 0.999, 0.999, 0.960, 0.957, and 0.986, respectively. Confusion matrices, failure cases, and reliability results are presented in the supplementary materials.

**Table 3:** Dental finding classification results. All metrics are reported as mean over 5 cross-validation models.

Dental finding	Support	Precision	Sensitivity	F1-score	Specificity	Accuracy	AUC
Caries lesion	688	0.6929	0.7241	0.7077	0.9667	0.9440	0.9598
Crown	384	0.9527	0.9125	0.9322	0.9975	0.9931	0.9974
Filling	1770	0.9353	0.9391	0.9371	0.9793	0.9696	0.9936
Implant	60	0.9618	0.9767	0.9686	0.9997	0.9995	0.9995
Pontic	81	0.9595	0.9457	0.9519	0.9995	0.9989	0.9973
Periapical lesion	96	0.5069	0.5375	0.5197	0.9929	0.9869	0.9574
Primary tooth	301	0.9804	0.9927	0.9865	0.9991	0.9989	0.9994
Retained root	66	0.8331	0.8212	0.8261	0.9985	0.9969	0.9860
Root canal treatment	479	0.9845	0.9775	0.9809	0.9989	0.9975	0.9997
Unerupted tooth	570	0.9638	0.9653	0.9644	0.9969	0.9945	0.9989

AUC = area under receiver-operating-characteristics (ROC) curve.

**Fig. 2:**
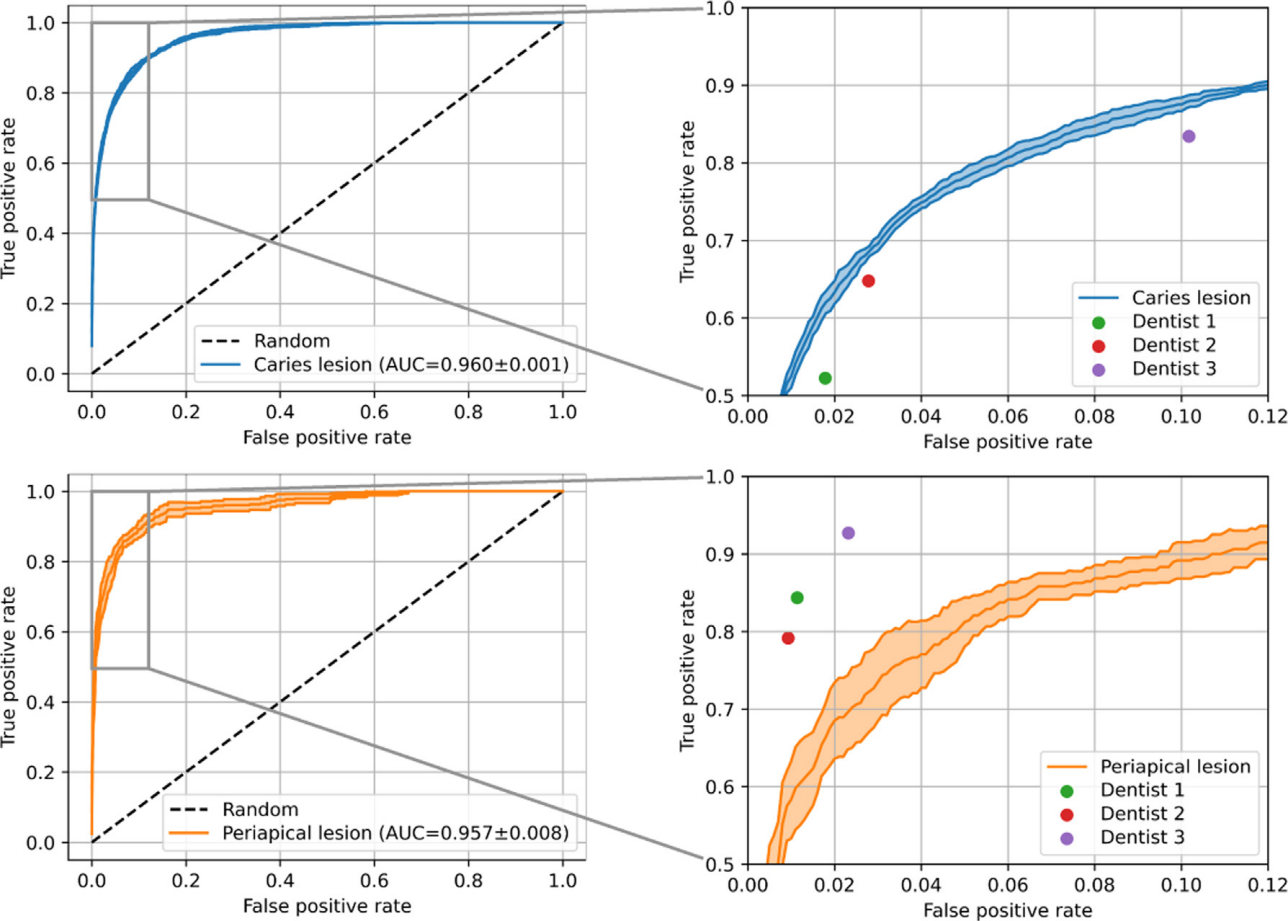
Receiver-operating-characteristics (ROC) curves for caries lesions and periapical lesions. The dashed line represents a random classifier with an accuracy of 0.5. Each ROC curve is shown with the middle line and outer lines representing the mean and mean ± standard deviation across 5 cross-validation models. The panels on the right show an enlarged view of the top-left quadrant of each ROC curve, as well as the operating points of 3 dentists. AUC = area under the ROC curve.

Similarly, the dentists were also less accurate for caries lesions, PA lesions, and retained roots, with sensitivities below 0.9, compared to the other dental findings (Table 4). Comparing the F1-scores of the AI system to the dentists, the system was more effective for caries lesions, crowns, fillings, primary teeth, root canal treatments, and unerupted teeth. In contrast, the dentists were more effective for implants, pontics, PA lesions, and retained roots. Investigating the results for caries lesions further, the AI system was reliably more effective compared to all 3 dentists (Figure 2). Conversely, results for PA lesions were less reliable and the AI system was less effective compared to all dentists.

**Table 4:** Comparison of dental finding classification between AI models and dentists for the held-out radiographs.

Finding	Reader	Prec.	Sens.	F1	Spec.	Finding	Reader	Prec.	Sens.	F1	Spec.
Caries lesion	Model	**0.693**	**0.724**	**0.708**	**0.967**	Periapical lesion	Model	**0.507**	0.538	0.520	**0.993**
	Dentist	0.640	0.668	0.629	0.951		Dentist	0.459	**0.854**	**0.589**	0.985
Crown	Model	**0.953**	0.913	**0.932**	**0.998**	Primary tooth	Model	**0.980**	**0.993**	**0.986**	**0.999**
	Dentist	0.935	**0.915**	0.924	0.996		Dentist	0.979	0.928	0.953	**0.999**
Filling	Model	**0.935**	**0.939**	**0.937**	**0.979**	Retained root	Model	0.833	0.821	0.826	0.998
	Dentist	0.916	0.932	0.924	0.973		Dentist	**0.857**	**0.869**	**0.862**	**0.999**
Implant	Model	0.962	0.977	0.969	**1.000**	Root canal treatment	Model	0.984	**0.977**	**0.981**	**0.999**
	Dentist	**0.989**	**0.989**	**0.989**	**1.000**		Dentist	**0.987**	0.965	0.976	**0.999**
Pontic	Model	0.959	**0.946**	0.952	**1.000**	Unerupted tooth	Model	0.964	**0.965**	**0.964**	0.997
	Dentist	**0.974**	0.941	**0.957**	**1.000**		Dentist	**0.981**	0.940	0.960	**0.999**

The model metrics are reported as mean over 5 cross-validation models and the dentist metrics are reported as mean over 3 dentists. Better metrics for each dental finding are highlighted. Prec. = Precision, Sens. = Sensitivity, F1 = F1-score, Spec. = Specificity.

**Fig. 3:**
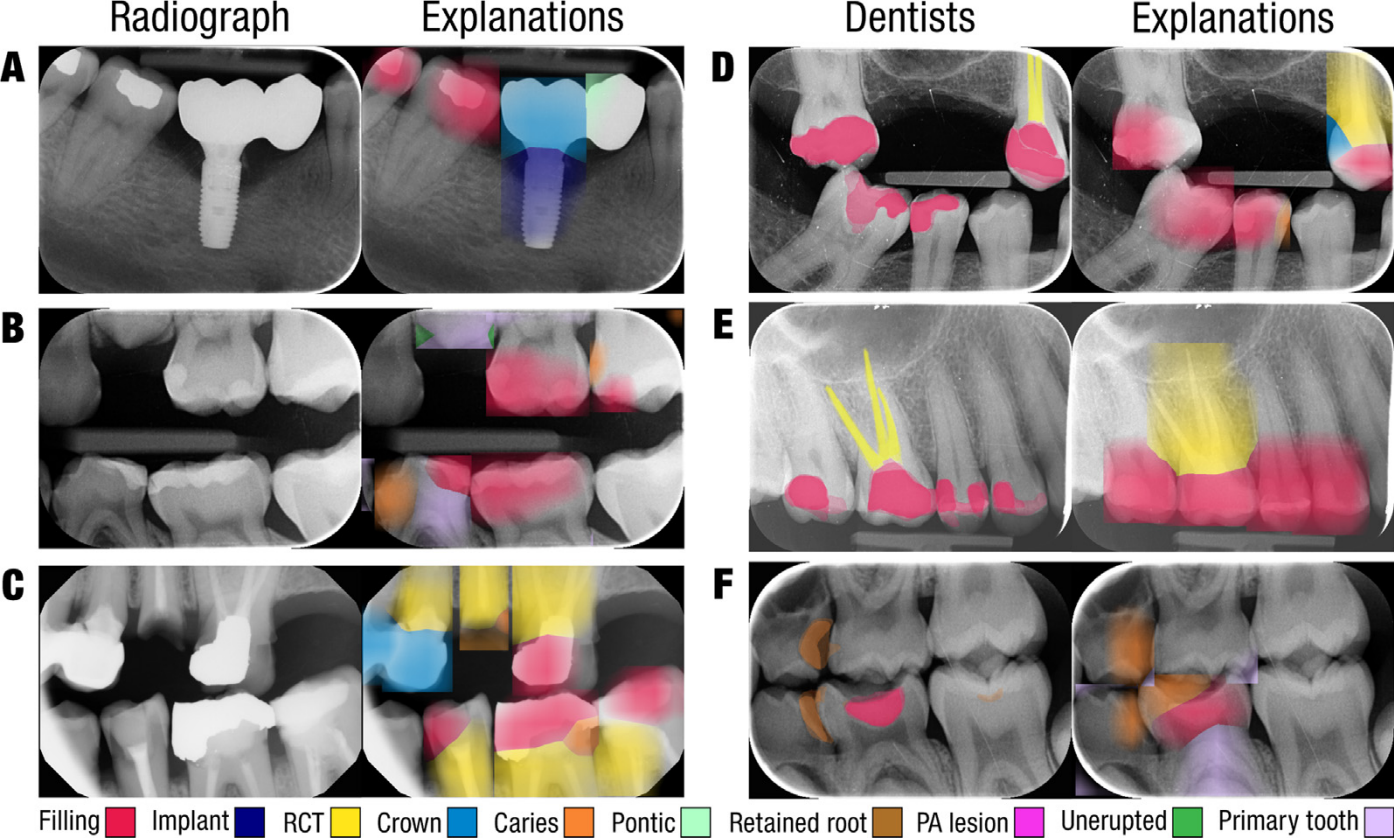
(A-C) Grad-CAM explanations of dental findings in held-out intraoral radiographs. Predicted teeth in each intraoral radiograph were processed by the dental finding classifiers to determine the presence or absence of ten dental findings. For each present finding, the corresponding Grad-CAM was superimposed on the radiograph with a finding-specific color, see the legend at the bottom. To improve readability, each Grad-CAM was limited to the tooth’s predicted bounding box and colors were not mixed. (D-F) Comparison of dentist segmentations and Grad-CAM explanations for held-out intraoral radiographs. Three dentists segmented fillings, periapical (PA) lesions, root canal treatments (RCTs), and caries lesions, which are visualized with color maps where the intensity of the color represents the number of dentists that segmented the pixel.

Figure 3A-C shows the Grad-CAM explanations of the AI system for intraoral radiographs. This qualitative result provides an overview of the predicted dental findings. Lastly, Figure 3D-F shows an overlap between dentist segmentations and model explanations, revealing that the models used the same areas of interest to assess the dental findings.

## Discussion

This study aimed to develop a deep learning system for automated interpretation of dental radiographs and to evaluate it against expert annotations and dental practitioners. A 3-stage model was proposed combining modality classification (extraoral, intraoral), tooth detection and FDI numbering, and dental finding classification. Results showed promising effectiveness, with less effective results for the detection of primary teeth and the assessment of retained roots and teeth with caries lesions or periapical lesions.

An effective system for automated dental charting would alleviate the large time burden of dental practitioners to document and monitor each patient’s dental condition. Furthermore, automated assessments of dental findings related to diseases can support clinicians during diagnosis, improving their consistency and diagnostic accuracy.^35^^,^^36^ When properly integrated into a patient management software, a dentist can acquire a dental radiograph and interpret it shortly after with AI-supported assessments. After revising or confirming the automated findings, the patient records can be updated automatically, speeding up documentation. Furthermore, the model explanations may help with patient communication to clarify a treatment plan. Integrating AI into routine dental practice could thus provide substantial benefits both from a clinician’s and a patient’s perspective.

When interpreting the results, it was observed that tooth detection in OPGs was more effective than in intraoral radiographs in terms of tooth F1-score and FDI accuracy and less effective in terms of mean average precision (mAP). The increased field of view of an OPG provides a large context for effective tooth detection and numbering. In contrast, intraoral radiographs have a higher resolution, making it easier to outline tooth bounding boxes precisely. Therefore, each modality offers unique advantages, which are reflected in the results (Table 2).

The lower effectiveness for detecting and numbering primary teeth compared to permanent teeth likely arose from both data- and task-specific factors. The dataset contained substantially fewer primary teeth (n = 3,700) than permanent teeth (n = 56,110), limiting the model’s ability to generalize. In addition, mixed dentitions pose greater challenges due to tooth overlap and the need to assess tooth morphology to distinguish between primary and permanent tooth variants at the same position in the dental arch. These challenges disproportionately affect the effectiveness for primary teeth, as some primary teeth are already lost by the time the first radiograph of a child is acquired. Given the high image resolution and attention-based Swin-B backbone architecture, the difference in effectiveness is most likely caused by data scarcity and the inherent challenges of mixed-dentition cases rather than model limitations.

Dental finding classification was more effective for findings unrelated to disease than for caries lesions, PA lesions, and retained roots, which corresponds to the uncertainty whether a dental finding is present or absent in Table 1. The study’s process of aggregating the assessments of independent clinicians to derive an aggregated reader score may not have resulted in a consistent confidence, and, by extension, may not have yielded a consistent reference for determining whether the dental finding was present (score ≥ 0.5). This is especially evident for uncertain dental findings, i.e. where clinicians disagree, resulting in reduced effectiveness.

The dental findings for which the system was less effective than the dentists occurred fewer than 1,000 times in all radiographs, whereas the other findings occurred at least 3,000 times. This relationship between sample size and effectiveness suggests that collecting and annotating more images would further increase effectiveness, possibly surpassing that of dentists for all ten dental findings.

A recent study investigated the diagnostic accuracy and consistency (Fleiss’ κ) of dental students for caries detection in bitewings with and without support from an AI system.^37^ Without support, the dental students achieved a sensitivity, specificity, F1-score, and κ of 0.679, 0.877, 0.664, and 0.590 respectively, showing an accuracy similar to the 3 dentists (Supplementary Table 7). Conversely, with support, the sensitivity and consistency increased significantly to 0.790 and 0.702, respectively. The developed AI system should thus serve as a supporting tool for the interpretation of dental radiographs, to benefit from both the clinician’s experience and the system’s examination of all tooth surfaces.

Diagnosis of caries lesions in OPGs is considered unreliable, especially initial enamel lesions.^38^^,^^39^ Therefore, this study only considered moderate and severe caries lesions in OPGs. The results for caries lesion diagnosis (OPG F1 = 0.730, bitewing F1 = 0.667, PA F1 = 0.747; Supplementary Table 7) may thus not be representative of initial caries diagnosis in OPGs.

The largest difference between the AI system and the dentists was observed for the sensitivity of PA lesions (Table 4; system = 0.54, dentists = 0.85). This may be due to the radiographic characteristics of a PA lesion being removed when preparing a single-tooth crop, as the tooth bounding box often did not include the full extension of the lesion. Assessing a tooth for the presence of a PA lesion without this crucial region would be more challenging, leading to the reduced sensitivity. Furthermore, the sensitivity was higher for PAs than OPGs (Supplementary Table 7), indicating that the higher resolution and improved contrast of PAs may also benefit the model predictions.

Several works on the automated interpretation of dental radiographs have been published. Kabir et al. developed a deep learning system for the detection, segmentation, and numbering of teeth in OPGs and intraoral radiographs.^40^ Their system processed all radiographs of a patient simultaneously and utilized teeth from the OPG to arrange the radiographs in a full-mouth series, thereby optimizing FDI numbering.

Görürgöz et al. developed an automated method for tooth detection and numbering in PAs.^41^ 1686 PAs were collected to develop a deep learning system comprising a jaw classification model and region-specific detection models. Results showed an F1-score of 0.872, which is lower than the F1-score of 0.978 achieved by this study for intraoral radiographs.

Pinheiro et al. developed a tooth segmentation model for permanent and primary teeth in OPGs.^42^ The model achieved a mean average precision (mAP) of 0.807 and 0.678 for permanent and primary teeth, respectively. In contrast, the AI system achieved mAPs of 0.875 and 0.625 for these 2 categories, respectively. Thus, the developed system finds primary tooth detection in OPGs still challenging, which may be due to the small proportion of primary teeth in OPGs (2,257 out of 45,750).

Yurttaş et al. proposed an AI model for diagnostic charting on OPGs.^43^ 1084 OPGs were included, and ten dental findings were annotated by 2 specialist radiologists with bounding boxes and labels. The study developed a dental finding detection model, achieving effective results for crowns (F1 = 0.91), pontics (F1 = 0.82), RCTs (F1 = 0.82), implants (F1 = 0.94), unerupted teeth (F1 = 0.86), and fillings (F1 = 0.87) and less effective results for retained roots (F1 = 0.74) and caries lesions (F1 = 0.38). The current system was more effective for each dental finding, likely due to the increased number of radiographs.

Lastly, Oztekin et al. manually cropped teeth from OPGs and developed classification models to predict the presence of caries for each single-tooth crop.^44^ Additionally, the model’s predictions were explained by superimposing Grad-CAMs on the original tooth crops and inserting those into the original OPG, similar to Figure 3, which provides an overview of areas where the model predicted caries. Furthermore, these areas were compared to expert segmentations, showing high agreement between the model and experts, as also shown in Figure 3D-F.

While previous studies reported different results, direct comparisons with the current study should be made cautiously. Variations in model design, data quantity and quality, and evaluation procedures substantially influence outcomes. Limited public access to dental radiograph datasets further hinders reproducibility and fair performance comparisons.

Although this study included several radiograph types, one limitation is that some radiograph types, such as occlusal and pediatric radiographs, were underrepresented (<100 radiographs). Furthermore, the radiographs were collected using convenience sampling, which could have introduced a selection bias. In addition, the reference standard of the dental findings in the held-out radiographs could be improved. A dental finding was deemed present when aggregated reader scores exceeded 0.5, which were determined based on at least 4 clinicians using the Dawid-Skene model.^27^ This model determined the reliability of each clinician based on the total dataset and weighted the assessments of more reliable clinicians higher when computing the aggregated reader scores, thereby promoting assessments of more experienced clinicians, as they are reported to be more reliable.^45^ Despite this resulting in a confident score for most dental findings, the presence of a caries lesion, PA lesion, or retained root was uncertain for some teeth. These scores were included during training to stimulate model calibration and during evaluation to better represent the reliability of the models and dentists compared to the clinicians. Another limitation is that the model explanations are no substitute for a pixel-wise segmentation of each dental finding. Though the explanations provide a comprehensive overview of the detected findings by highlighting their general locations, the explanations lack in precision to gain a deeper understanding of the model’s decision-making.

This study can be extended by collecting more challenging radiographs, such as those with a mixed dentition or those with uncommon dental findings, including root resorption, periodontal bone loss, and supernumerary teeth, to expand the capabilities of the AI system. Alternatively, radiographs could be collected from a third clinical center to perform an external validation of the developed AI system. Moreover, a prospective investigation into the utility of the system for efficiency and diagnostic accuracy during routine dental care can be performed. Where the current study compared the automated system to dentists, this potential work could compare dentists who use the system to those who do not.

Finally, the developed system is capable of diagnosis of dental radiographs. However, determining an informed treatment plan often relies on a series of radiographs over time. For example, if a caries lesion is progressing toward the pulp, a dentist is recommended to perform restorative treatment; however, no treatment is necessary if no progression of the caries lesion can be observed. Developing an automated system for proposing treatment options thus requires longitudinal data. Future work could focus on collecting dental radiographs from multiple time points to facilitate longitudinal monitoring and informed treatment recommendations.

## Conclusions

A deep learning system for interpreting dental radiographs outperformed dentists in identifying common dental findings, while showing reduced effectiveness for rare conditions. Developing reliable and generalizable diagnostic models requires large-scale data collection and expert annotation, especially for uncommon dental findings. With sufficient high-quality data and rigorous validation, such AI systems are expected to substantially improve diagnostic accuracy and efficiency.

## Funding

This research was supported by the Radboud Dental AI Hub and partially funded by AID s.r.o. This research did not receive any specific grant from funding agencies in the public, commercial, or not-for-profit sectors.

## Data availability statement

The collected radiographs and annotations will not be made available.

## Author contributions

**Niels van Nistelrooij:** Writing – original draft, Investigation, Methodology, Software, Validation, Visualization. **Peter Jurkáček:** Writing – review and editing, Conceptualization, Data curation, Resources. **Julian Runge:** Writing – review and editing, Data curation, Validation. **Wesley Do:** Writing - review and editing, Data curation, Validation. **Khalid El Ghoul:** Writing - review and editing, Data curation, Validation. **Tong Xi:** Writing - review and editing, Validation, Supervision. **Maximiliano Sergio Cenci:** Writing - review and editing, Validation, **Bas A.C. Loomans:** Writing - review and editing, Validation, Project administration. **Shankeeth Vinayahalingam:** Writing - review and editing, Conceptualization, Funding acquisition, Investigation, Methodology, Supervision. All authors gave their final approval and agree to be accountable for all aspects of the work.

## Conflict of interest

The authors declare the following financial interests/personal relationships which may be considered as potential competing interests: Shankeeth Vinayahalingam reports financial support was provided by AID s.r.o. Niels van Nistelrooij reports a relationship with Ardim B.V. that includes: employment. Shankeeth Vinayahalingam reports a relationship with Ardim B.V. that includes: board membership. If there are other authors, they declare that they have no known competing financial interests or personal relationships that could have appeared to influence the work reported in this paper.

## Appendix Supplementary materials

Image, application 1

## Footnotes

Supplementary material associated with this article can be found in the online version at doi:10.1016/j.identj.2026.109576.
