# Supplementary material for: Development and Validation of AI System for Tooth Detection and Diagnosis in Dental Radiographs
Source: Int Dent J. 2026 Apr 26;76(4):109576. doi: 10.1016/j.identj.2026.109576 (PMC13134024; doi:10.1016/j.identj.2026.109576)
Supplement: Supplementary file 1 [file mmc1.docx]

Development and Validation of AI System for Tooth Detection and Diagnosis in Dental Radiographs

Niels van Nistelrooij, Peter Jurkáček, Julian Runge, Wesley Do, Khalid El Ghoul, Tong Xi, Maximiliano Sergio Cenci, Bas A.C. Loomans, Shankeeth Vinayahalingam

# 1. Model training

Training and inference were performed on a workstation with an RTX A6000 48 GB and 128 GB memory.

### Modality classification

This stage was implemented using the EfficientNetV2 architecture (Tan and Le 2021) provided by MMPreTrain (v. 1.1.1; MMPreTrain Contributors 2023) based on PyTorch 2.1.2 (Paszke et al. 2019). The AdamW optimizer was used with a base learning rate of 0.001 and a weight decay of 0.05 (Loshchilov and Hutter 2019). The learning rate was changed following a cosine annealing schedule with linear warmup. Data augmentations were applied by resizing, cropping, flipping, as well as several spatial and intensity augmentations (Isensee et al. 2021). Images of each modality were sampled equally often (Shen et al. 2016) and the label smoothing loss was applied for regularization (Szegedy et al. 2016). The model parameters were initialized by pre-training on the ImageNet dataset (Deng et al. 2009). Training was performed for a maximum of 80 epochs with a batch size of 256 and the final checkpoint for evaluation was selected using early stopping based on the F1-score for the radiographs from the validation fold.

### Tooth detection

The detection models were implemented using the RTMDet architecture (Lyu et al. 2022) with a Swin-B backbone (Liu et al. 2021) provided by MMDetection (v. 3.3.0; Chen et al. 2019) based on PyTorch 2.1.2 (Paszke et al. 2019). The same optimizer was used with a learning rate of 0.0005 and linear warmup. Data augmentations included resizing, cropping, and flipping, and input images were padded to make them square. The model parameters were initialized by first pre-training the backbone on the ImageNet dataset (Deng et al. 2009) and successively pre-training the full model on the COCO (Lin et al. 2014) and DENTEX challenge (Hamamci et al. 2023a, Hamamci et al. 2023b) datasets. Training was performed for a maximum of 75 epochs, where the learning rate was decreased by a factor of 10 for the final 10 epochs. A batch size of 2 was used and radiographs with rare FDI numbers were oversampled to address class imbalance (Yaman et al. 2023). The final parameters were determined using an exponential moving average of the checkpoints (Tarvainen and Valpola 2017) and early stopping based on the mean average precision for the modality-specific radiographs from the validation fold.

### Dental finding classification

The training strategy of the modality classifier was used again with a few changes. Data augmentation retained the original bounding box in the input image during cropping to ensure that image characteristics used to determine the presence of a dental finding were always present. A model was supervised using the cross-entropy loss with the aggregated reader score as target. Compared to label smoothing used for the modality classifier, the aggregated scores can promote generalizability by calibrating the model’s confidence more effectively, thus preventing overfitting (Szegedy et al. 2016).

# 2. Tooth detection

The qualitative results in Supplementary Figure 1 and Supplementary Figure 2 show representative tooth detection results. The system could detect non-supported crowns as teeth and errors were observed for significantly overlapping teeth in OPGs. Moreover, the qualitative results on intraoral radiographs show some confusion on whether to include teeth at the margin, resulting in a false-positive and false-negative prediction compared to the annotations.

Two held-out radiographs where tooth detection failed are presented in Supplementary Figure 3. The first case shows braces and a blurred anterior region, resulting in missed maxillary anterior teeth. The second case includes a rotated intraoperative periapical radiograph, where all teeth were missed. The OPG and PA failure cases may be addressed with data augmentation, by simulating low-quality OPGs and by introducing random orthogonal rotations, respectively. Furthermore, the current models can be extended with tooth segmentation to predict a more precise outline of the predicted teeth.

# 3. Dental finding classification

The confusion matrices in Supplementary Figure 4 show the diverse range of prevalences of the dental findings, in particular fillings and implants.

As an illustrative example, four teeth with aggregated reader scores of 0.24, 0.54, 0.74, and 1.0 are shown in Supplementary Figure 5, demonstrating large differences between the aggregated reader scores and model scores.

# References

Chen K, Wang J, Pang, J, Cao Y, Xiong Y, Li X, Sun S, Feng W, Liu Z, Xu J, et al. 2019. MMDetection: Open

MMLab Detection Toolbox and Benchmark. [accessed 2025 Apr 13].

<https://doi.org/10.48550/arXiv.1906.07155>.

Deng J, Dong W, Socher R, Li L, Li K, Fei-Fei L. 2009. ImageNet: A large-scale hierarchical image database.

In: Proceedings of the IEEE Conference on Computer Vision and Pattern Recognition (CVPR).

IEEE. p. 248-255.

Hamamci IE, Er S, Simsar E, Sekuboyina A, Gundogar M, Stadlinger B, Mehl A, Menze B. 2023a. Diffusion

Based Hierarchical Multi-Label Object Detection to Analyze Panoramic Dental X-rays. [accessed

2025 Apr 13]. <https://doi.org/10.48550/arXiv.2303.06500>.

Hamamci IE, Er S, Simsar E, Yuksel AE, Gultekin S, Ozdemir SD, Yang K, Li HB, Pati S, Stadlinger B, et al.

2023b. DENTEX: An Abnormal Tooth Detection with Dental Enumeration and Diagnosis

Benchmark for Panoramic X-rays. [accessed 2025 Apr 13].

<https://doi.org/10.48550/arXiv.2305.19112>.

Isensee F, Jaeger PF, Kohl SAA, Petersen J, Maier-Hein KH. 2021. nnU-Net: a self-configuring

method for deep learning-based biomedical image segmentation. *Nat Methods.* 18(1):203-211.

Lin T, Maire M, Belongie S, Hays J, Perona P, Ramanan D, Dollár P, Zitnick CL. 2014. Microsoft COCO:

Common Objects in Context. In: Proceedings of the 13th Europen Conference on Computer

Vision (ECCV). Springer. p. 740-755.

Liu Z, Lin Y, Cao Y, Hu H, Wei Y, Zhang Z, Lin S, Guo B. 2021. Swin Transformer: Hierarchical Vision

Transformer using Shifted Windows. In: Proceedings of the IEEE/CVF International Conference

on Computer Vision (ICCV). IEEE. p. 10012-10022.

Loshchilov I, Hutter F. 2019. Decoupled weight decay regularization. In: The Seventh International

Conference on Learning Representations. ICLR.

Lyu C, Zhang W, Huang H, Zhou Y, Wang Y, Liu Y, Zhang S, Chen K. 2022. RTMDet: An Empirical Study of

Designing Real-Time Object Detectors. [accessed 2025 Apr 13].

<https://doi.org/10.48550/arXiv.2212.07784>.

MMPreTrain Contributors. 2023. OpenMMLab's Pre-training Toolbox and Benchmark. [accessed 2025

Apr 13]. <https://github.com/open-mmlab/mmpretrain>.

Paszke A, Gross S, Massa F, Lerer A, Bradbury J, Chanan G, Killeen T, Lin Z, Gimelshein N, Antiga L, et al.

2019. PyTorch: an imperative style, high-performance deep learning library. In: Proceedings of

the 32^nd^ International Conference on Neural Information Processing Systems. Curran Associates

Inc. p. 8024-8035.

Shen L, Lin Z, Huang Q. 2016. Relay Backpropagation for Effective Learning of Deep Convolutional Neural

Networks. In: Proceedings of the 14th Europen Conference on Computer Vision (ECCV).

Springer. p. 467-482.

Szegedy C, Vanhoucke V, Ioffe S, Schlens J, Wojna Z. 2016. Rethinking the Inception Architecture for

Computer Vision. In: Proceedings of the IEEE Conference on Computer Vision and Pattern

Recognition (CVPR). IEEE. p. 2818-2826.

Tan M, Le Q. 2021. EfficientNetV2: Smaller Models and Faster Training. In: Proceedings of the 38^th^

International Conference on Machine Learning. PMLR. p. 10096–10106.

Tarvainen A, Valpola H. 2017. Mean teachers are better role models: Weight-averaged consistency

targets improve semi-supervised deep learning results. In: Proceedings of the 31st International

Conference on Neural Information Processing Systems. Curran Associates Inc. p. 1195-1204.

Yaman B, Mahmud T, Liu C. 2023. Instance-Aware Repeat Factor Sampling for Long-Tailed Object

Detection. [accessed 2025 Apr 13]. <https://doi.org/10.48550/arXiv.2305.08069>.

**Supplementary Table 1.** Definitions of ten dental findings.

| **Finding** | **Definition** |
| --- | --- |
| Caries lesion | We utilize classification based on Radiographic staging.  Radiographic staging:  ● 0 = No radiolucency.  RA Initial stages:  ● RA 1 = radiolucency in the outer ½ of the enamel.  ● RA 2 = radiolucency in the inner ½ of the enamel ± EDJ (enamel-dentin junction).  ● RA 3 = radiolucency limited to the outer 1/3 of dentin.  RB Moderate stages:  ● RB 4 = radiolucency reaching the middle 1/3 of dentin.  RC Extensive stages:  ● RC 5 = radiolucency reaching the inner 1/3 of dentin, clinically cavitated.  ● RC 6 = radiolucency into the pulp, clinically cavitated).  Primary vs Secondary decays:  ● Primary = Dental caries involving a discrete area of tooth not including an existing restoration.  ● Secondary = Recurrent dental caries involving a discrete area of a tooth including an area adjacent to an existing restoration or sealant. |
| Crown | Dental crowns are prosthetic restorations that are placed over a damaged or weakened tooth to restore its shape, strength, and function. They are custom-made caps that cover the visible portion of the tooth above the gingiva line, providing stability and improving aesthetics. |
| Filling | A dental filling is a procedure to repair a decayed or damaged tooth by removing the affected area and filling it with a durable material, restoring the tooth's function and appearance. The filling material is shaped and polished to match the tooth’s natural contour during the procedure. |
| Implant | Dental implants are advanced prosthetics that replace missing teeth. They consist of a titanium post implanted in the alveolar bone, an abutment that connects to the post, and a custom-made crown that looks and functions like a natural tooth. Dental implants offer improved function and aesthetics.  ● Peri-implantitis:  Peri-implantitis is an inflammatory condition that affects the tissues surrounding a dental implant. Similar to periodontal disease but specific to implants, it involves the inflammation of the gums (peri-implant mucosa) and can lead to bone loss around the implant. Peri-implantitis can compromise the stability and longevity of the implant if not promptly diagnosed and treated.  ● Vertical bone loss:  Vertical bone loss around dental implants refers to the reduction of bone height along the implant's sides. This can occur due to various factors, including poor oral hygiene, inflammation, infection, or biomechanical issues. Significant vertical bone loss can weaken the implant's support and compromise its stability.  ● Endosteal:  These are the most common type of dental implants and are placed directly into the alveolar bone. They consist of a titanium post that serves as an artificial tooth root, onto which a crown is attached.  ● Subperiosteal:  Placed on or above the jawbone but beneath the gum tissue, these implants are suitable for patients with insufficient bone height or density for traditional implants.  ● Single Tooth:  Used to replace a single missing tooth, a single tooth implant involves an implant post and a crown that matches the adjacent teeth.  ● Implant-Supported Bridge:  Multiple Teeth Implant involves using implants to support a bridge that replaces multiple adjacent missing teeth.  ● All-on-6 or All-on-4:  These concepts involve using only four or six strategically placed implants to support a full arch of replacement teeth, making them a cost-effective solution for full-mouth rehabilitation.  ● Zygomatic:  Utilized when there is inadequate upper jawbone, zygomatic implants anchor into the cheekbone (zygoma) to support a full arch of teeth or overdenture. |
| Pontic | A pontic is defined as an artificial tooth on a fixed dental prosthesis that replaces a missing natural tooth, restoring its function and esthetics. It usually fills the space previously occupied by the clinical crown of the missing tooth. |
| Periapical lesion | A periapical lesion refers to a localized abnormality or change in the bone and surrounding tissues at the tip (apex) of a tooth's root. It can result from various factors, such as infections, inflammation, or trauma, and is often visible on dental X-rays.  ● Periapical granuloma:  A periapical granuloma is a localized inflammatory lesion that forms at the apex (tip) of a tooth's root in response to chronic infection or irritation. It is often a result of bacterial infection that spreads from the tooth's pulp (innermost part) into the surrounding bone and tissues.  ● Radicular cyst:  A radicular cyst, also known as a periapical cyst, is a common type of odontogenic cyst that forms near the apex (tip) of a tooth's root. It arises as a result of chronic inflammation or infection within the tooth's pulp (innermost part) that spreads to the surrounding tissues and bone.  ● Caused by retained root:  A periapical lesion caused by a retained root refers to an inflammatory condition affecting the area around the apex (tip) of a tooth root that remains in the jaw after a tooth has been partially removed or broken off. This condition is commonly observed in dental practice and often results from incomplete tooth extraction, trauma, or decay that leads to the fracture of the tooth near the gum line, leaving the root or a fragment of it behind.  ● Periapical abscess:  A periapical abscess has features similar to periapical granulomas and periapical cysts with a varying degree of peripheral cortication, which makes it difficult to distinguish them from one another. Cortical erosion or perforation seen in CBCT examinations and the presence of edema might provide additional information in distinguishing abscesses; however, the early stages of periapical abscesses often do not present with these characteristics. |
| Primary tooth | Deciduous teeth, commonly known as baby teeth or primary teeth, are the first set of teeth that erupt in the mouth during early childhood. |
| Retained root | Retained dental roots arise most commonly due to prior dental extraction procedures, during which they are intentionally or unintentionally left behind. They can also arise from trauma that results in dental fractures. Their removal may be warranted to make way for a future dental implant or dental prosthesis or due to odontogenic pain, infection, or associated pathology. |
| Root canal treatment | The root canal obturation is a dental procedure used to treat infection located in the pulp cavity of a tooth. Root canal treatment is needed when dental X-rays show that the pulp is invaded by a bacterial infection. When bacteria invade the pulp, it can become inflamed, allowing the infection to spread further within the tooth and surrounding tissues.. This treatment involves the removal of infected or inflamed pulp tissue, disinfection of the root canal system, and sealing the space to prevent reinfection.  ● Homogenous:  A successful homogeneous root canal treatment involves the complete removal of infected or damaged pulp tissue, thorough cleaning and disinfection of the root canals, proper shaping to accommodate the filling material, and sealing the canals to prevent reinfection.  ● Non homogenous:  In a non-homogeneous treatment, the root canal system is not consistently cleaned, shaped, or sealed throughout its full length. Certain areas may be inadequately treated, leaving behind residual infection or unfilled canal spaces, which can compromise the success of the procedure and lead to clinical complications. |
| Unerupted tooth | Refers to a tooth that has not yet erupted through the gingiva into the oral cavity. This term is often used to describe teeth that are still developing and have not fully emerged into their proper position in the dental arch. |

**Supplementary Table 2.** Characteristics of ten dental findings for each radiograph type, including orthopantomograms (OPGs), bitewings (BWs), periapical radiographs (PA), and other radiographs. The count and prevalence per image (Prev.) are determined for aggregated reader scores of at least 0.5. The confidence (Conf.) is determined as the average aggregated reader score of at least 0.1. ‘-‘ denotes that this finding was not present for a certain radiograph type.

| **Finding** | **Type** | **Count** | **Prev. (%)** | **Conf.** |  | **Finding** | **Type** | **Count** | **Prev. (%)** | **Conf.** |
| --- | --- | --- | --- | --- | --- | --- | --- | --- | --- | --- |
| Caries lesion | OPG | 3498 | 68 | 0.739 |  | Periapical lesion | OPG | 598 | 24 | 0.698 |
|  | BW | 1777 | 55 | 0.868 |  |  | BW | 1 | 0.066 | 1.00 |
|  | PA | 247 | 35 | 0.861 |  |  | PA | 44 | 9.3 | 0.728 |
|  | Other | 237 | 72 | 0.909 |  |  | Other | 2 | 0.5 | 0.620 |
| Crown | OPG | 2386 | 37 | 0.969 |  | Primary tooth | OPG | 2257 | 15 | 0.990 |
|  | BW | 785 | 25 | 0.959 |  |  | BW | 896 | 14 | 0.997 |
|  | PA | 335 | 43 | 0.963 |  |  | PA | 45 | 2.9 | 1.000 |
|  | Other | 3 | 1.6 | 1.000 |  |  | Other | 502 | 97 | 1.000 |
| Filling | OPG | 8019 | 79 | 0.971 |  | Retained root | OPG | 444 | 14 | 0.898 |
|  | BW | 5393 | 85 | 0.982 |  |  | BW | 15 | 0.66 | 0.943 |
|  | PA | 720 | 68 | 0.975 |  |  | PA | 9 | 1.6 | 0.909 |
|  | Other | 69 | 26 | 0.990 |  |  | Other | 0 | 0 | - |
| Implant | OPG | 324 | 8.2 | 1.000 |  | Root canal treatment | OPG | 2765 | 52 | 0.996 |
|  | BW | 209 | 10 | 0.998 |  |  | BW | 797 | 33 | 0.991 |
|  | PA | 104 | 18 | 1.000 |  |  | PA | 414 | 61 | 0.998 |
|  | Other | 0 | 0 | - |  |  | Other | 3 | 1.6 | 0.793 |
| Pontic | OPG | 585 | 16 | 0.999 |  | Unerupted tooth | OPG | 4625 | 40 | 0.988 |
|  | BW | 152 | 7.3 | 0.999 |  |  | BW | 242 | 13 | 0.985 |
|  | PA | 71 | 13 | 1.000 |  |  | PA | 46 | 6.0 | 1.000 |
|  | Other | 1 | 0.54 | 0.980 |  |  | Other | 128 | 40 | 1.000 |

**Supplementary Table 3.** Characteristics of nine dental findings for primary (Prim.) and permanent (Perm.) teeth. The count and prevalence (Prev.) are determined for aggregated reader scores of at least 0.5. The confidence (Conf.) is determined as the average aggregated reader score of at least 0.1. ‘-‘ denotes that this finding was not present for any primary or permanent tooth.

| **Finding** | **Teeth** | **Count** | **Prev. (%)** | **Conf.** |  | **Finding** | **Teeth** | **Count** | **Prev. (%)** | **Conf.** |
| --- | --- | --- | --- | --- | --- | --- | --- | --- | --- | --- |
| Caries lesion | Perm. | 4793 | 8.3 | 0.775 |  | Periapical lesion | Perm. | 633 | 1.1 | 0.703 |
|  | Prim. | 966 | 26 | 0.830 |  |  | Prim. | 12 | 0.32 | 0.573 |
| Crown | Perm. | 3459 | 6.0 | 0.966 |  | Retained root | Perm. | 458 | 0.79 | 0.916 |
|  | Prim. | 50 | 1.3 | 0.951 |  |  | Prim. | 10 | 0.27 | 0.556 |
| Filling | Perm. | 13601 | 24 | 0.975 |  | Root canal treatment | Perm. | 3969 | 6.9 | 0.996 |
|  | Prim. | 600 | 16 | 0.977 |  |  | Prim. | 10 | 0.27 | 0.798 |
| Implant | Perm. | 637 | 1.1 | 0.999 |  | Unerupted tooth | Perm. | 5038 | 8.7 | 0.989 |
|  | Prim. | 0 | 0 | - |  |  | Prim. | 3 | 0.081 | 0.662 |
| Pontic | Perm. | 808 | 1.4 | 0.999 |  |  |  |  |  |  |
|  | Prim. | 1 | 0.027 | 0.980 |  |  |  |  |  |  |

**Supplementary Table 4.** Characteristics of ten dental findings based on gender. The count and prevalence per image (Prev.) are determined for aggregated reader scores of at least 0.5. The confidence (Conf.) is determined as the average aggregated reader score of at least 0.1.

| **Finding** | **Gender** | **Count** | **Prev. (%)** | **Conf.** |  | **Finding** | **Gender** | **Count** | **Prev. (%)** | **Conf.** |
| --- | --- | --- | --- | --- | --- | --- | --- | --- | --- | --- |
| Caries lesion | Male | 3307 | 60 | 0.770 |  | Periapical lesion | Male | 438 | 14 | 0.701 |
|  | Female | 2452 | 57 | 0.802 |  |  | Female | 207 | 8.1 | 0.698 |
| Crown | Male | 1971 | 33 | 0.966 |  | Primary tooth | Male | 2234 | 17 | 0.992 |
|  | Female | 1538 | 30 | 0.967 |  |  | Female | 1466 | 17 | 0.994 |
| Filling | Male | 7148 | 75 | 0.976 |  | Retained root | Male | 385 | 9.2 | 0.906 |
|  | Female | 7053 | 80 | 0.975 |  |  | Female | 83 | 2.9 | 0.873 |
| Implant | Male | 357 | 9.6 | 1.000 |  | Root canal treatment | Male | 2166 | 42 | 0.994 |
|  | Female | 280 | 9.7 | 0.999 |  |  | Female | 1813 | 43 | 0.996 |
| Pontic | Male | 502 | 13 | 1.000 |  | Unerupted tooth | Male | 3244 | 26 | 0.987 |
|  | Female | 307 | 8.9 | 0.997 |  |  | Female | 1797 | 23 | 0.992 |

**Supplementary Table 5.** Reliability of tooth detection and numbering metrics across five cross-validation models. Range and standard deviation (SD) are reported.

|  |  | **Tooth F1** | | **FDI Accuracy** | | **mAP@50-95** | |
| --- | --- | --- | --- | --- | --- | --- | --- |
| **Modality (n)** | **Teeth** | **Range** | **SD** | **Range** | **SD** | **Range** | **SD** |
| Extraoral (n=200) | Permanent | 0.991-0.992 | < 10^-3^ | 0.980-0.982 | < 10^-3^ | 0.873-0.877 | 0.001 |
|  | Primary | 0.932-0.960 | 0.009 | 0.974-0.982 | 0.003 | 0.614-0.644 | 0.010 |
|  | All | 0.989-0.990 | < 10^-3^ | 0.980-0.982 | < 10^-3^ | 0.774-0.786 | 0.004 |
| Intraoral (n=200) | Permanent | 0.978-0.980 | 0.001 | 0.952-0.964 | 0.005 | 0.853-0.867 | 0.005 |
|  | Primary | 0.947-0.977 | 0.010 | 0.892-0.969 | 0.028 | 0.879-0.905 | 0.009 |
|  | All | 0.977-0.980 | 0.001 | 0.952-0.964 | 0.005 | 0.864-0.877 | 0.005 |

**
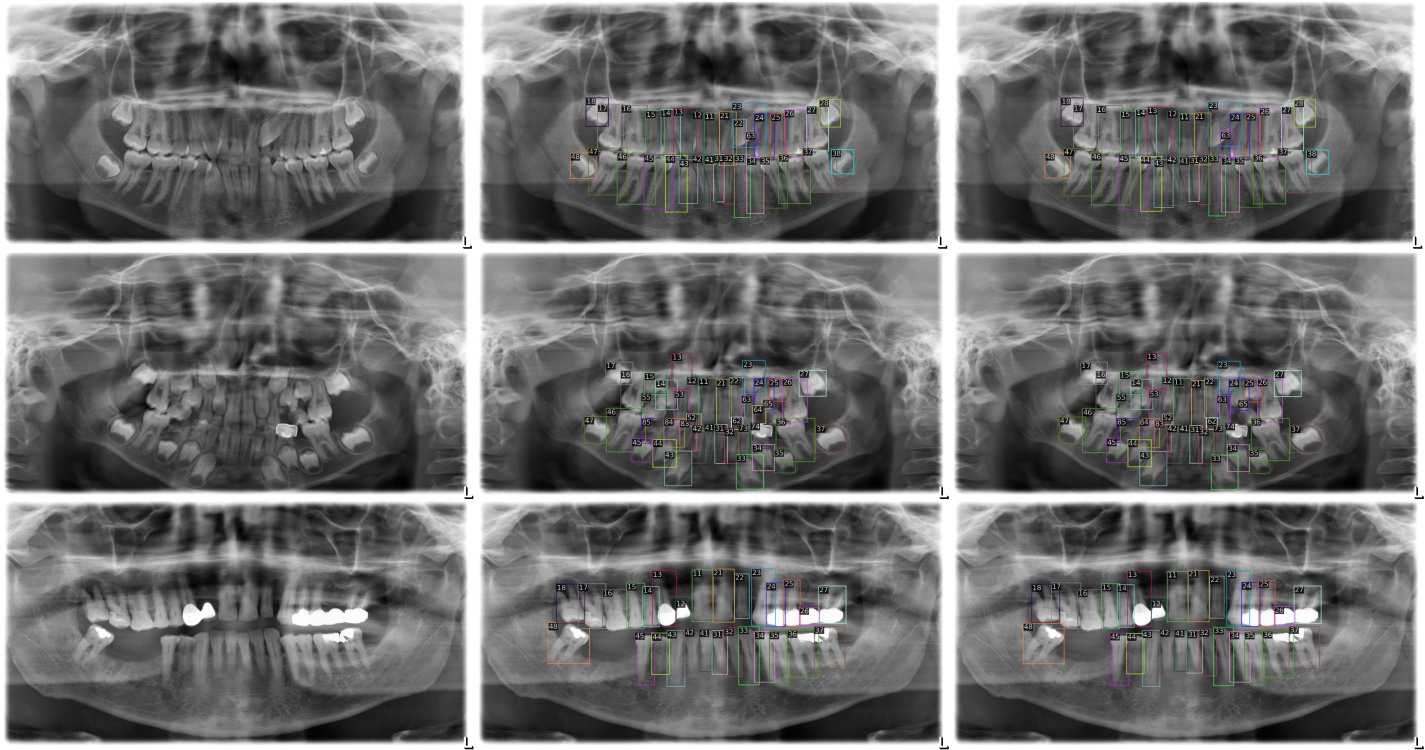
**

**Supplementary Figure 1.** Qualitative results of tooth detection on OPGs. Three OPGs from held-out patients are shown with the ground-truth annotations and model predictions in the middle and right columns, respectively. Results from the model trained on the most effective cross-validation split are shown.

**
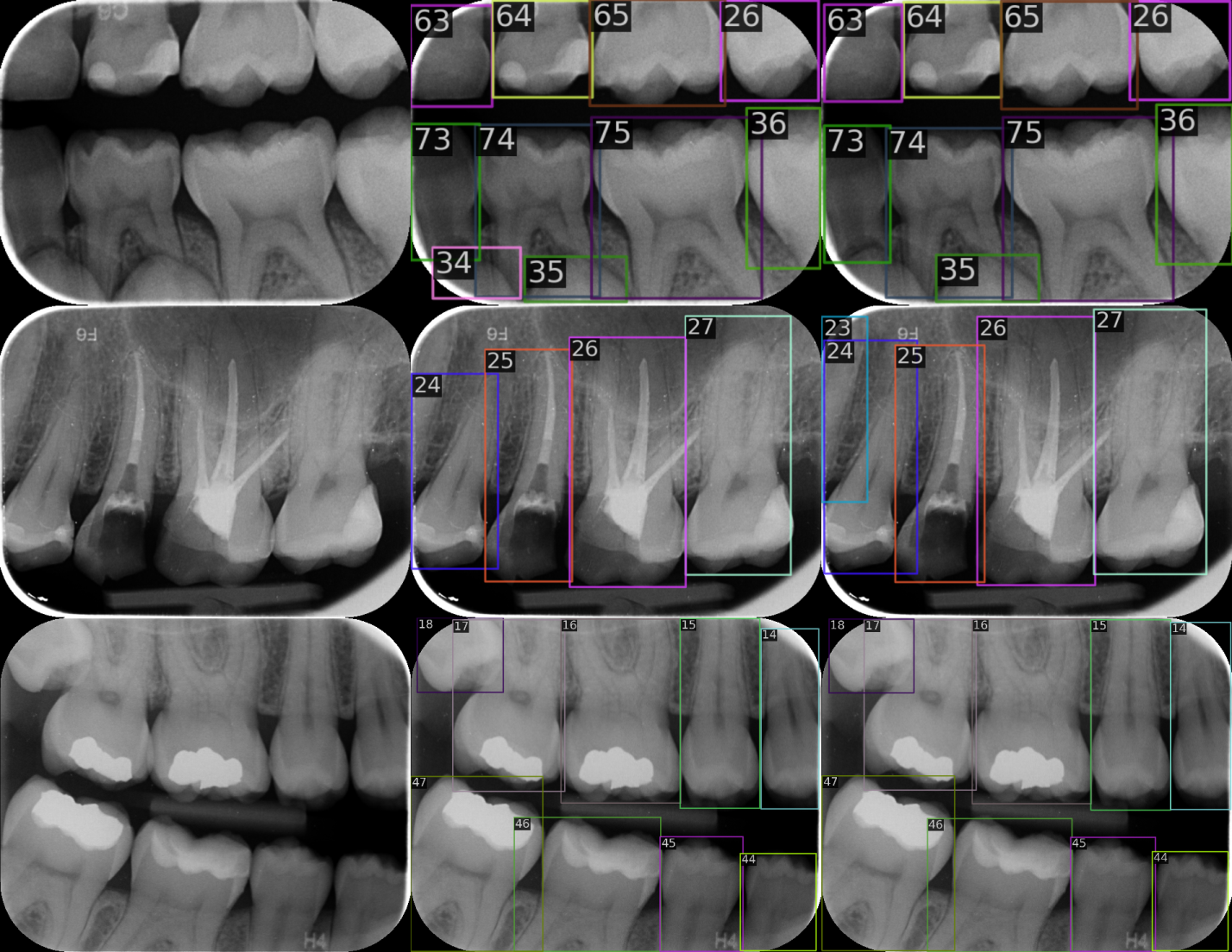
**

**Supplementary Figure 2.** Qualitative results of tooth detection on intraoral dental radiographs. Two bitewings and a PA from held-out patients are shown with the ground-truth annotations and model predictions in the middle and right columns, respectively. Results from the model trained on the most effective cross-validation split are shown.

**
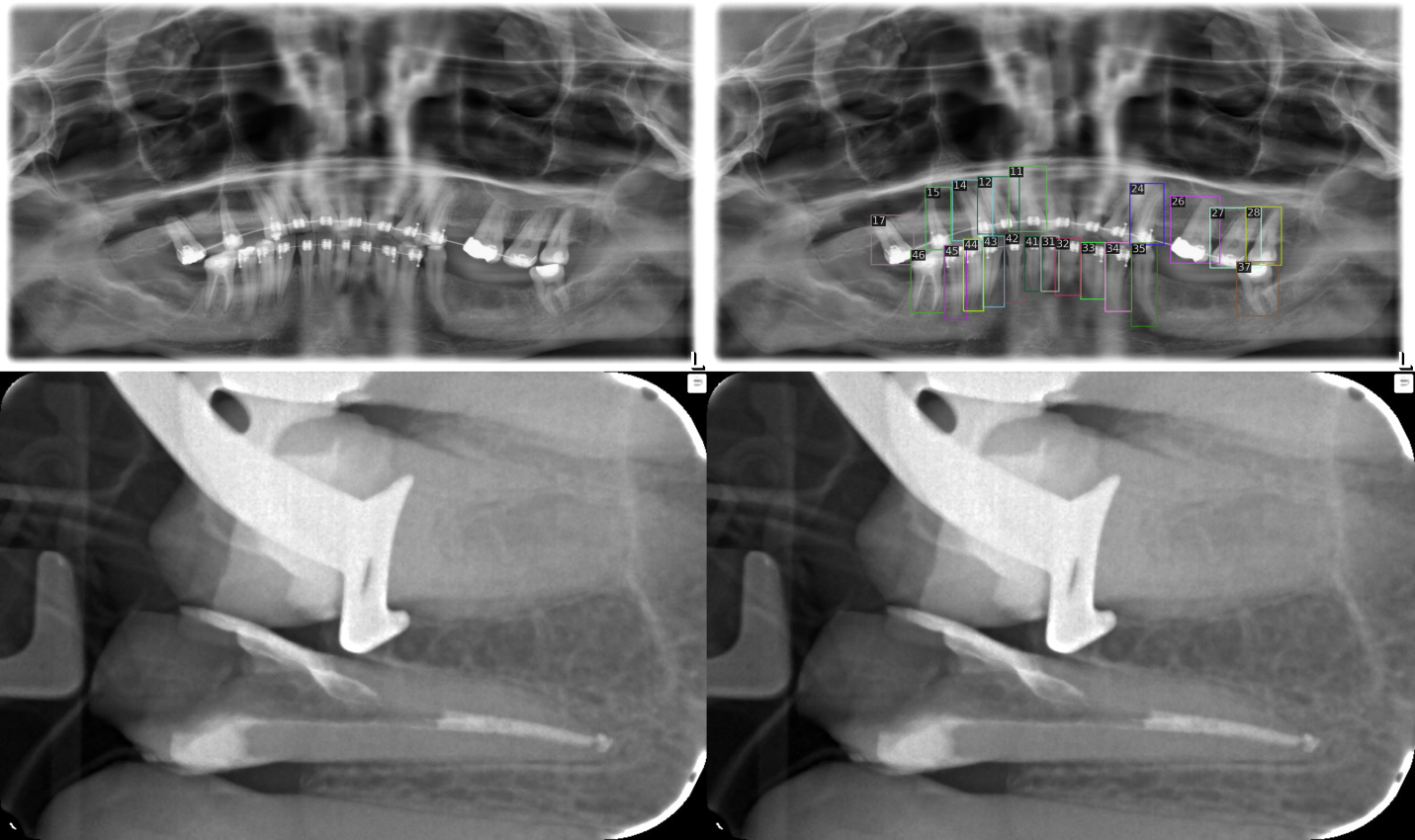
**

**Supplementary Figure 3.** Tooth detection failure cases. The left column shows the original radiograph and the right column shows the model predictions.

|  | **Precision** | | **Sensitivity** | | **F1-score** | | **Specificity** | |
| --- | --- | --- | --- | --- | --- | --- | --- | --- |
| **Dental finding** | **Range** | **SD** | **Range** | **SD** | **Range** | **SD** | **Range** | **SD** |
| Caries lesion | 0.661-0.716 | 0.018 | 0.688-0.747 | 0.021 | 0.701-0.716 | 0.006 | 0.960-0.972 | 0.004 |
| Crown | 0.946-0.964 | 0.006 | 0.898-0.919 | 0.008 | 0.925-0.939 | 0.004 | 0.997-0.998 | < 10^-3^ |
| Filling | 0.921-0.953 | 0.010 | 0.927-0.946 | 0.007 | 0.932-0.940 | 0.003 | 0.974-0.985 | 0.004 |
| Implant | 0.909-1.000 | 0.031 | 0.917-1.000 | 0.031 | 0.940-1.000 | 0.021 | 0.999-1.000 | < 10^-3^ |
| Pontic | 0.885-1.000 | 0.041 | 0.914-0.963 | 0.018 | 0.917-0.981 | 0.021 | 0.999-1.000 | < 10^-3^ |
| Periapical lesion | 0.447-0.596 | 0.064 | 0.479-0.573 | 0.032 | 0.462-0.573 | 0.039 | 0.991-0.995 | 0.002 |
| Primary tooth | 0.971-0.987 | 0.006 | 0.990-0.997 | 0.002 | 0.982-0.990 | 0.003 | 0.999-0.999 | < 10^-3^ |
| Retained root | 0.784-0.887 | 0.033 | 0.773-0.879 | 0.038 | 0.797-0.859 | 0.021 | 0.998-0.999 | < 10^-3^ |
| Root canal treatment | 0.977-0.989 | 0.004 | 0.967-0.987 | 0.007 | 0.975-0.985 | 0.004 | 0.998-0.999 | < 10^-3^ |
| Unerupted tooth | 0.952-0.984 | 0.011 | 0.954-0.974 | 0.006 | 0.961-0.969 | 0.003 | 0.996-0.999 | 0.001 |

**Supplementary Table 6.** Reliability of dental finding classification metrics across five cross-validation models. Range and standard deviation (SD) are reported.


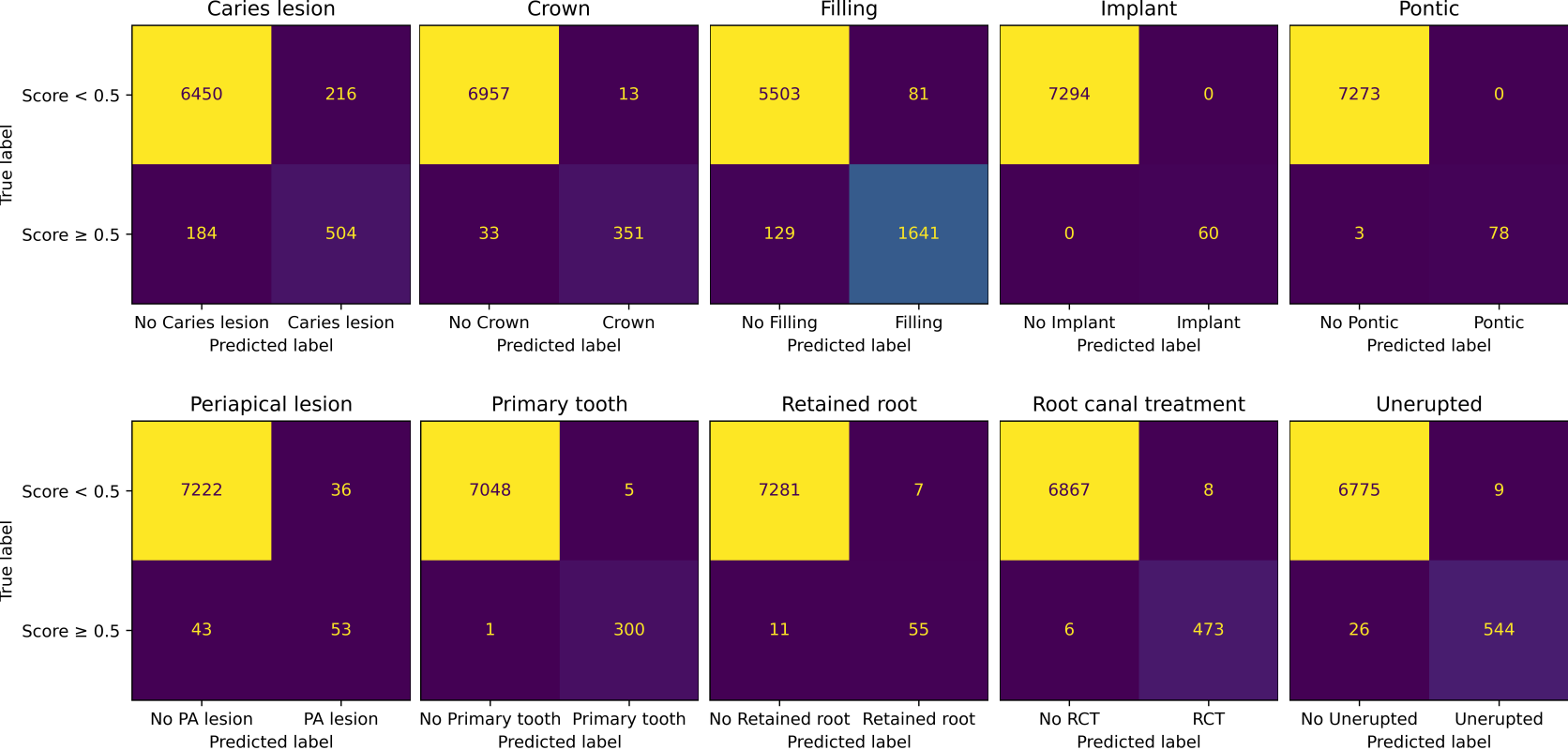


**Supplementary Figure 4.** Confusion matrices of results for the held-out patients. The confusion matrix of the most effective cross-validation model is shown for each dental finding. A cell color represents the value of that cell with a brighter color for a larger value.

**Supplementary Table 7.** Comparison of dental finding classification on the held-out radiographs for different radiograph types. The metrics are reported as mean over five cross-validation models. ‘-‘ denotes that this finding was not present for a certain radiograph type. OPG = orthopantomogram, BW = bitewing, PA = periapical, Prec. = Precision, Sens. = Sensitivity, F1 = F1-score, Spec. = Specificity.

| **Finding** | **Type** | **Prec.** | **Sens.** | **F1** | **Spec.** |  | **Finding** | **Type** | **Prec.** | **Sens.** | **F1** | **Spec.** |
| --- | --- | --- | --- | --- | --- | --- | --- | --- | --- | --- | --- | --- |
| Caries lesion | OPG | 0.705 | 0.758 | 0.730 | 0.972 |  | Periapical lesion | OPG | 0.509 | 0.550 | 0.527 | 0.991 |
|  | BW | 0.720 | 0.622 | 0.667 | 0.961 |  |  | BW | - | - | - | - |
|  | PA | 0.759 | 0.770 | 0.747 | 0.927 |  |  | PA | 0.447 | 0.650 | 0.407 | 0.967 |
| Crown | OPG | 0.954 | 0.931 | 0.942 | 0.998 |  | Primary tooth | OPG | 0.979 | 0.999 | 0.989 | 0.999 |
|  | BW | 0.942 | 0.868 | 0.902 | 0.997 |  |  | BW | 0.990 | 0.987 | 0.988 | 1.000 |
|  | PA | 0.993 | 0.910 | 0.949 | 0.999 |  |  | PA | 0.960 | 0.800 | 0.871 | 0.999 |
| Filling | OPG | 0.924 | 0.922 | 0.923 | 0.982 |  | Retained root | OPG | 0.833 | 0.822 | 0.827 | 0.998 |
|  | BW | 0.969 | 0.967 | 0.968 | 0.976 |  |  | BW | 1.000 | 1.000 | 1.000 | 1.000 |
|  | PA | 0.930 | 0.951 | 0.940 | 0.947 |  |  | PA | - | - | - | - |
| Implant | OPG | 0.976 | 0.980 | 0.977 | 1.000 |  | Root canal treatment | OPG | 0.983 | 0.985 | 0.984 | 0.999 |
|  | BW | 0.985 | 0.967 | 0.975 | 1.000 |  |  | BW | 0.992 | 0.980 | 0.986 | 1.000 |
|  | PA | 0.978 | 1.000 | 0.988 | 0.999 |  |  | PA | 0.975 | 0.970 | 0.972 | 0.992 |
| Pontic | OPG | 0.976 | 0.953 | 0.964 | 1.000 |  | Unerupted tooth | OPG | 0.963 | 0.968 | 0.965 | 0.996 |
|  | BW | 0.899 | 1.000 | 0.945 | 0.999 |  |  | BW | 0.971 | 0.970 | 0.970 | 1.000 |
|  | PA | 1.000 | 0.857 | 0.923 | 1.000 |  |  | PA | 1.000 | 1.000 | 1.000 | 1.000 |

**Supplementary Table 8.** Comparison of dental finding classification on the held-out radiographs for primary (Prim.) and permanent (Perm.) teeth. The metrics are reported as mean over five cross-validation models. ‘-‘ denotes that this finding was not present for any primary or permanent tooth. Prec. = Precision, Sens. = Sensitivity, F1 = F1-score, Spec. = Specificity.

| **Finding** | **Teeth** | **Prec.** | **Sens.** | **F1** | **Spec.** |  | **Finding** | **Teeth** | **Prec.** | **Sens.** | **F1** | **Spec.** |
| --- | --- | --- | --- | --- | --- | --- | --- | --- | --- | --- | --- | --- |
| Caries lesion | Perm. | 0.696 | 0.723 | 0.708 | 0.969 |  | Periapical lesion | Perm. | 0.510 | 0.549 | 0.526 | 0.993 |
|  | Prim. | 0.763 | 0.732 | 0.742 | 0.940 |  |  | Prim. | 0.363 | 0.800 | 0.445 | 0.975 |
| Crown | Perm. | 0.952 | 0.912 | 0.932 | 0.997 |  | Retained root | Perm. | 0.834 | 0.841 | 0.837 | 0.998 |
|  | Prim. | 1.000 | 1.000 | 1.000 | 1.000 |  |  | Prim. | 0.900 | 0.700 | 0.733 | 0.999 |
| Filling | Perm. | 0.937 | 0.937 | 0.937 | 0.980 |  | Root canal treatment | Perm. | 0.984 | 0.978 | 0.981 | 0.999 |
|  | Prim. | 0.936 | 0.973 | 0.954 | 0.983 |  |  | Prim. | 1.000 | 1.000 | 1.000 | 1.000 |
| Implant | Perm. | 0.962 | 0.977 | 0.969 | 1.000 |  | Unerupted tooth | Perm. | 0.964 | 0965 | 0.964 | 0.997 |
|  | Prim. | - | - | - | - |  |  | Prim. | 1.000 | 1.000 | 1.000 | 1.000 |
| Pontic | Perm. | 0.964 | 0.946 | 0.954 | 1.000 |  |  |  |  |  |  |  |
|  | Prim. | - | - | - | - |  |  |  |  |  |  |  |

**Supplementary Table 9.** Comparison of dental finding classification on the held-out radiographs for both genders. The metrics are reported as mean over five cross-validation model. Prec. = Precision, Sens. = Sensitivity, F1 = F1-score, Spec. = Specificity.

| **Finding** | **Gender** | **Prec.** | **Sens.** | **F1** | **Spec.** |  | **Finding** | **Gender** | **Prec.** | **Sens.** | **F1** | **Spec.** |
| --- | --- | --- | --- | --- | --- | --- | --- | --- | --- | --- | --- | --- |
| Caries lesion | Male | 0.687 | 0.735 | 0.710 | 0.964 |  | Periapical lesion | Male | 0.533 | 0.585 | 0.554 | 0.992 |
|  | Female | 0.754 | 0.683 | 0.717 | 0.978 |  |  | Female | 0.497 | 0.458 | 0.467 | 0.995 |
| Crown | Male | 0.998 | 0.941 | 0.953 | 0.998 |  | Primary tooth | Male | 0.979 | 0.989 | 0.984 | 0.999 |
|  | Female | 0.942 | 0.879 | 0.909 | 0.997 |  |  | Female | 0.986 | 0.998 | 0.992 | 0.999 |
| Filling | Male | 0.937 | 0.932 | 0.935 | 0.984 |  | Retained root | Male | 0.830 | 0.825 | 0.827 | 0.998 |
|  | Female | 0.944 | 0.939 | 0.941 | 0.977 |  |  | Female | 0.875 | 0.818 | 0.842 | 1.000 |
| Implant | Male | 0.987 | 0.963 | 0.974 | 1.000 |  | Root canal treatment | Male | 0.979 | 0.985 | 0.982 | 0.999 |
|  | Female | 0.979 | 0.993 | 0.986 | 1.000 |  |  | Female | 0.980 | 0.984 | 0.982 | 0.998 |
| Pontic | Male | 0.983 | 0.942 | 0.962 | 1.000 |  | Unerupted tooth | Male | 0.955 | 0.958 | 0.956 | 0.996 |
|  | Female | 0.915 | 0.982 | 0.946 | 0.999 |  |  | Female | 0.988 | 0.980 | 0.984 | 0.999 |


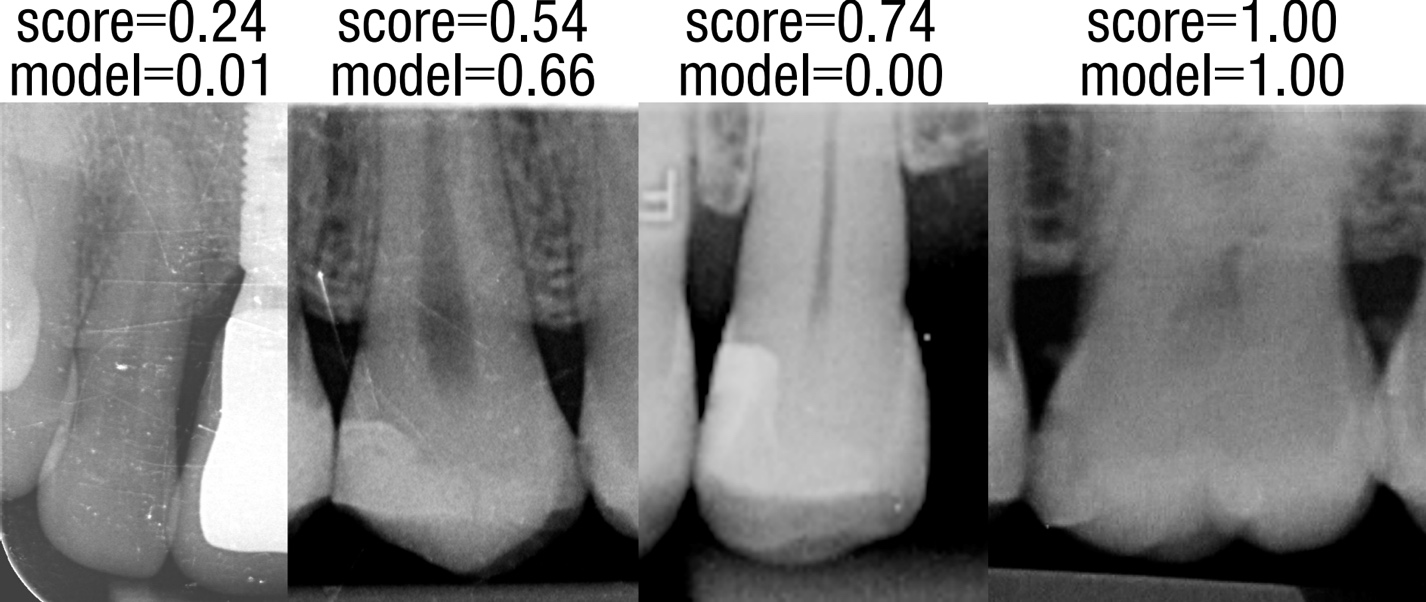


**Supplementary Figure 5.** Four teeth and their corresponding aggregated reader scores and model confidences for the presence of a caries lesion.
